# *Cladonia* lichens on extensive green roofs: evapotranspiration, substrate temperature, and albedo

**DOI:** 10.12688/f1000research.2-274.v2

**Published:** 2014-01-23

**Authors:** Amy Heim, Jeremy Lundholm

**Affiliations:** 1 Biology Department, Saint Mary's University, Halifax, B3H 3C3, Canada

## Abstract

Green roofs are constructed ecosystems that provide ecosystem services in urban environments. Shallow substrate green roofs subject the vegetation layer to desiccation and other environmental extremes, so researchers have evaluated a variety of stress-tolerant vegetation types for green roof applications. Lichens can be found in most terrestrial habitats.  They are able to survive extremely harsh conditions, including frequent cycles of desiccation and rehydration, nutrient-poor soil, fluctuating temperatures, and high UV intensities. Extensive green roofs (substrate depth <20cm) exhibit these harsh conditions, making lichens possible candidates for incorporation into the vegetation layer on extensive green roofs.  In a modular green roof system, we tested the effect of *Cladonia* lichens on substrate temperature, water loss, and albedo compared to a substrate-only control. Overall, the *Cladonia* modules had significantly cooler substrate temperatures during the summer and significantly warmer temperatures during the fall.  Additionally, the *Cladonia* modules lost significantly less water than the substrate-only control. This implies that they may be able to benefit neighboring vascular plant species by reducing water loss and maintaining favorable substrate temperatures.

## Introduction

Green roofs are constructed ecosystems, designed to provide ecosystem services such as the reduction of heat flux through the roof, the capture of storm water, and the provision of habitat for animals ^1, 2^. Green roofs consist of a vegetation and growing medium layer (substrate) over engineered layers that provide a root barrier, drainage, and/or water retention layers ^1^. The majority of green roofs constructed in temperate climates are “extensive” green roofs, characterized by shallow growing media (<20cm), which minimizes the weight added to the building. Shallow growing media in such systems have led to a reliance on succulent plant species in the vegetation layer, to ensure survival during drought conditions. Other plant growth forms have also been used on extensive green roofs, with grasses, forbs, and mosses among the most frequent ^3, 4^. Lichens are often found on conventional roof surfaces ^5^ and form an important component of cryptogamic crusts in terrestrial ecosystems ^6^. Cryptogamic crusts in arid environments perform a range of ecosystem services, such as stabilizing soil, fixing nitrogen, and enhancing soil water holding capacity ^7^, but have not been intentionally planted in green roof ecosystems.

Lichens are symbiotic organisms, an association between a fungus (the mycobiont) and one or more algal and/or cyanobacterial photobionts ^8, 9^. Typically the mycobiont forms 95% of the lichen body. They are the dominant plant life in harsh environments such as the Arctic, Antarctic, mountains, and dry land crusts ^8^. Lichen species are widespread and can be found from the Arctic to deserts; they can survive frequent cycles of desiccation and rehydration, nutrient-poor soil, fluctuating temperatures, and UV light intensities ^8, 10^. They can survive and grow on the bare surface of rocks and in poor soils such as heathlands, peat lands, sand dunes, and toxic spoil heaps ^10^. Selection of vegetation types appropriate for extensive green roofs often involves identifying local habitats that have characteristics in common with green roofs (shallow soil, harsh abiotic conditions) ^11^; lichens are a common component of rock barrens, dunes, and heathland habitats that can be a source of plants for green roofing projects ^12, 13^.

Lichens are lightweight and can be found growing naturally on bare tile or slate rooftops ^1^. This could make them a candidate for roofs on buildings with low weight-loading capabilities. Species of the genus *Cladonia,* large fruticose lichens that colonize bare soils, are common in cold temperate climates. These lichens produce bundles of hyphae that stabilize the soil and add organic matter. The light color of the lichen can reflect solar radiation, keeping the soil cool and moist ^14^. These characteristics indicate the possible utility of *Cladonia* as a component of extensive green roofs. In this study, the performance of key green roof ecosystem functions in green roof modules planted with live *Cladonia* lichens was compared with substrate-only controls.

## Methods

**Figure 1.**
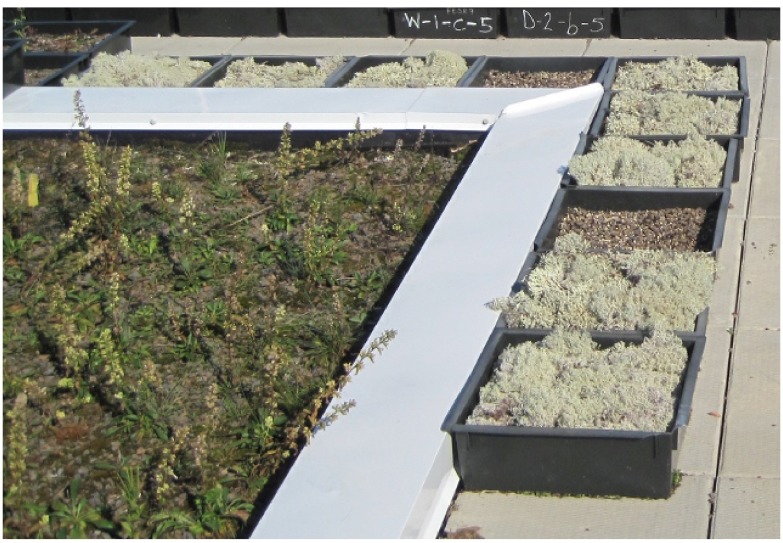
Placement of lichen trial on the east side of the roof of the five-story Atrium building at Saint Mary’s University in Halifax, Nova Scotia, Canada (44°39′N, 63°35′W).

The study site was located on the east side of the roof of the five-story Atrium building at Saint Mary’s University in Halifax, Nova Scotia, Canada (44°39′N, 63°35′W ) ( Figure 1). Data on soil temperature and water loss were collected between July and October 2012. Albedo was measured in September 2013. During the 2012 study period, the weather station on the adjacent green roof testing facility (~50m away from the study site) recorded the minimum monthly temperature as 6.7–20.7°C and the monthly maximum as 12–30°C. The monthly precipitation recorded from the green roof weather station averaged between 1.7 and 11.59mm. Albedo was collected on September 28, 2013 (average temperature: 14.12°C, total precipitation: 0mm).

**Figure 2.**
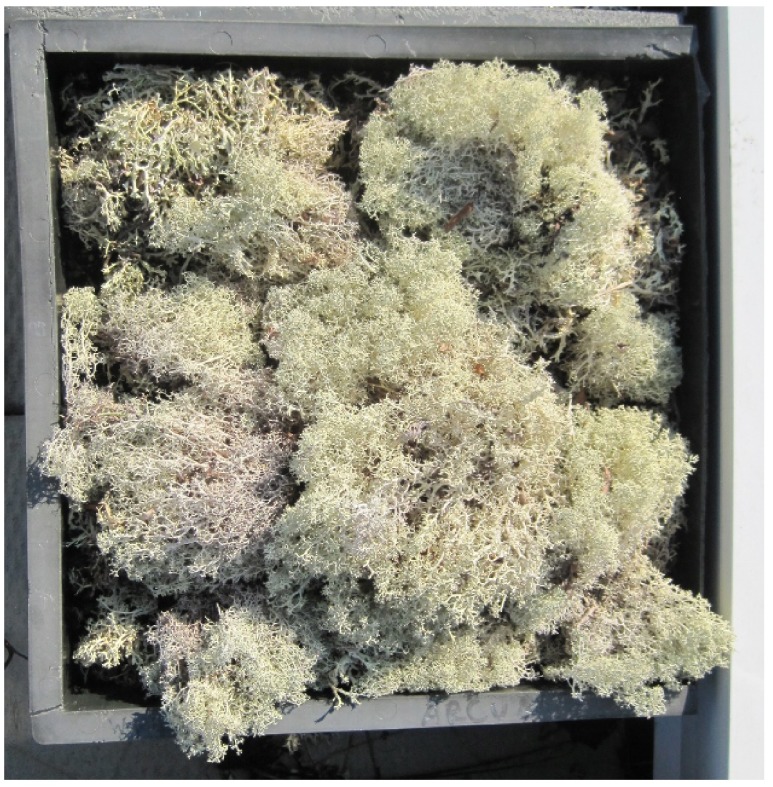
A *Cladonia* module used in the lichen trial. Each module had a length and width of 36cm with a substrate depth of 6cm.

In order to quantify the influence of *Cladonia* on green roof substrate, a trial was set up to determine the effects of *Cladonia* on soil temperature, water loss, and albedo. Ten green roof modules were placed on the Atrium roof on top of the roof surface, which was made up of grey concrete pavers. Each module had a length and width of 36cm, with a freely draining base (Polyflat ^®^, Stuewe & Sons Inc., Oregon, USA). Modules contained a root barrier/water retention fleece (length and width 36cm) over the base (EnkaRetain and Drain 3111 ^®^, Colbond Inc., North Carolina, USA) with 6cm depth of Sopraflor X substrate, purchased in 2011 (Soprema Inc., Drummondville, QC, Canada), over the root barrier/water retention layer. A soil test describing the composition of the Soprema X substrate can be seen in Supplementary Table 1. This experiment consisted of two substrate-only controls and eight modules covered 100% in *Cladonia* lichen approximately 6cm thick ( Figure 2). The lichen was collected from a coastal barrens site (Chebucto Head (44°30′N, 63°31′W)) in May 2012 and placed on the surface of the substrate. A mix of two lichen species, *C. terranova* and *C. boryi*, was used; both species have similar colors and growth forms, and co-occur in lichen mats. Lichens were alive when transplanted and shoot tips were marked to determine incremental growth over the experiment.

### Substrate temperature and volumetric water content (VWC)

Substrate temperature was recorded using a Taylor 9878 Slim-Line Pocket Thermometer Probe (Commercial Solutions Inc., Edmonton, Alberta, Canada) once a month throughout the growing season. The temperature was recorded from the center of each module approximately 2cm below the substrate surface when exposed to full sun, no more than two hours from solar noon. Only one measurement was recorded for each module on the day of measurement. These spot measurements represented maximal substrate temperatures and were collected during hot sunny conditions to provide maximum contrast between controls and planted green roof modules. This technique does not characterize the long-term provisioning of thermal benefits but has been used to contrast performance of different plant species or vegetation types during hot conditions ^15^. Volumetric water content of the substrate (VWC: water volume/soil volume × 100%) was recorded one day after a rain event and again one day later if no new showers were observed. Water loss was determined by subtracting the day one VWC from the day two VWC, which provides an index of net evapotranspiration over 24 hours ^15^. This variable is also used as an index of the hydrological performance of green roof systems. Measurements were taken once in August, September, and October 2012. VWC was measured using a GS3 probe with the ProCheck soil moisture control unit (Decagon Devices Inc., Pullman, Washington, USA) inserted into the center of each module adjacent to the target species to a depth of 5cm. Separate two sample t-tests were used to compare water loss and substrate temperature between control and lichen treatments. A p-value <0.05 was considered significant. All statistical analysis was analyzed using R project for statistical engineering version 3.0.1 ( http://www.r-project.org/index.html).

### Albedo

Albedo was not originally part of this trial, it was added later in order to get a better understanding of how *Cladonia* lichen could affect green roof substrates; visually, the lichens were very similar throughout both 2012 and 2013. Albedo was measured on September 28, 2013. The information was gathered from five lichen modules (100% cover) from the previous season and five substrate only controls created by removing vegetation from the old lichen modules (due to wind damage, only five lichen modules could be salvaged). Albedo was measured with a single LI-COR pyranometer sensor and LI-250A light meter (LI-COR Biosciences, Lincoln, Nebraska, United States) attached to a fixed position 35cm above the soil surface. The incoming solar radiation (Wm ^-2^) was measured by pointing the sensor 180° from the roof surface. The reflected radiation was measured by rotating the sensor 180° to face each module. Albedo was calculated by dividing the reflected radiation by the incoming radiation ^15^, and a one-tailed t-test was used to compare the control and lichen modules. A p-value <0.05 was considered significant.

## Results

The albedo of the *Cladonia* modules was significantly higher than the control modules ( Table 1, Data Set 1). Substrate temperatures for July and August were significantly lower in the *Cladonia* modules compared to the controls ( Table 1). In September, there was no significant difference in soil temperature between the *Cladonia* modules and the controls. In October, the *Cladonia* modules had significantly higher substrate temperatures than the controls ( Table 1, Data Set 2). Regarding water loss, there was no significant difference between the *Cladonia* modules and the control for August, but in September and October, *Cladonia* modules lost significantly less water than the controls ( Table 1, Data Set 3).

**Table 1.** Mean (± SE) temperature (°C), water loss (reduction in % VMC) and albedo (reflected radiation/incoming radiation) of the *Cladonia* modules compared to the substrate-only controls. An (*) indicates a significant difference (>0.05) between the substrate-only control and *Cladonia* treatment determined by a t-test.

Measurement	Month	Control	*Cladonia*	P-value
**Temperature**	July 2012*	35.90±1.700	30.64±0.604	0.0062
	August 2012*	31.05±0.250	26.46±0.397	0.0006
	September 2012	26.90±0.900	26.48±0.370	0.6314
	October 2012*	24.7±0.200	26.51±0.306	0.0227
**Water loss**	August 2012	3.350±0.650	1.825±1.131	0.5395
	September 2012*	6.350±1.750	0.975±0.478	0.0022
	October 2012*	8.90±2.300	1.212±0.725	0.0025
**Albedo**	September 2013	0.2965±0.012	0.3323±0.11	0.0278

**Data Set 1.** Raw albedo data for the lichen modules and substrate only controls taken on September 28, 2013. Albedo was calculated by dividing the reflected radiation (Wm ^-2^) by the incoming radiation (Wm ^-2^).

Treatment	Incoming	Reflected	Albedo
**Lichen**	307.9	116.7	0.379019
**Lichen**	308.1	117	0.379747
**Lichen**	314.3	115.91	0.368788
**Lichen**	309	113.1	0.366019
**Lichen**	312	119.1	0.381731
**Control**	290.1	93.95	0.323854
**Control**	298.3	97.72	0.32759
**Control**	299.9	91.97	0.306669
**Control**	304.3	94.7	0.311206
**Control**	306.1	102.06	0.33342

**Data Set 2.** Raw temperature data (°C) for the lichen modules and substrate only controls during the 2012 growing season (mm/dd/yyyy).

Treatment	7/1/2013	8/4/2013	9/12/2013	10/3/2013
**Lichen**	31.9	28.3	27.7	27.5
**Lichen**	28.8	25.9	27.8	27.3
**Lichen**	31.6	26.5	26.9	27.2
**Lichen**	30.9	26	26.7	26.9
**Lichen**	33.4	28.1	26.5	26.5
**Lichen**	29.3	25.3	26	26
**Lichen**	30.8	25.9	25.4	25.5
**Lichen**	28.4	25.7	24.8	25.2
**Control**	37.6	31.3	27.8	24.9
**Control**	34.2	30.8	26	24.5

**Data Set 3.** Raw volumetric water content data (%) for the lichen modules and substrate only controls during the 2012 growing season (mm/dd/yyyy).

Treatment	8/21/2012	8/22/2012	9/11/2012	9/12/2012	10/3/2012	10/4/2012
**Lichen**	32.6	27.7	34.4	34.4	31.9	30.3
**Lichen**	38.8	39.9	41.8	42.7	42.3	42.2
**Lichen**	37.2	37.7	41.5	41.3	42.7	42.4
**Lichen**	37.3	39.6	42.7	42.7	42.7	37.1
**Lichen**	29.8	28.8	42.5	39.9	42.5	42.3
**Lichen**	35	33.8	41.2	39	42.6	41.4
**Lichen**	36.4	30.9	41	39.9	40.5	41.8
**Lichen**	33.6	27.7	41	38.4	42.5	40.5
**Control**	24	21.3	34	25.9	35.7	24.5
**Control**	23.7	19.7	30.3	25.7	31	24.4

## Discussion

Compared to the substrate-only controls, the *Cladonia* modules were cooler during hot conditions and retained more moisture in the substrate. These cooler temperatures may have been a result of shading and higher albedo. Even though albedo was only measured in 2013, the *Cladonia* lichen always had a lighter coloring than the bare substrate. Lighter colors are associated with higher albedo. Likewise, the lichen cover resulted in less net evapotranspiration compared with the bare substrate treatment. Interestingly, the soil temperature during October (average air temperature on date of measurement: 17.9°C) was significantly warmer in the *Cladonia* modules compared to the controls. This implies that a *Cladonia* mat may help reduce heat loss during the winter and help cool the roof during the summer.

Plants surrounded by *Cladonia* may benefit from such temperature regulation (cooler temperatures in the summer, warmer temperatures in the winter) and greater water availability. Other studies have shown that plants with mat-forming growth can benefit less drought-tolerant species ^16^. In addition to this, *Cladonia* lichens do not appear detrimental to plant growth. ** Vascular plant species growing out of these lichen mats is a natural occurrence on the coastal barrens of Nova Scotia and, during the trial, seedlings of trees and grasses (carried in by the wind or by birds) were observed growing out of these modules, although control modules also had such seedling growth. Some lichen species also play a key role in soil development in conditions of high abiotic stress ^8, 17^. If lichen inclusion on a green roof could improve the substrate properties for neighboring species, the entire system would benefit.

In this study, while the lichens remained in the roof modules for the duration of the experiment, it is not clear that the lichens remained alive. Fruticose lichens grow an average of 1.5–10mm per year ^14^. However, the relative growth rate of transplanted *Cladonia* mats compared to established *Cladonia* mats can besignificantly lower ^18^. The growth of *Cladonia* in these modules was undetectable. Additionally, during the 2012 growing season the *Cladonia* lichen did not suffer from wind damage and appeared equivalent in size as to when initially established. However, during the 2013 growing season the *Cladonia* lichen mats fragmented, decreasing in size (personal observation). This fragmentation led to wind damage that only occurred during the 2013 growing season. Since green roofs can be exposed to high winds, unattached lichen could decrease any value the lichen may have to offer. This fragmentation could have been due to a number of factors such as incompatible soil chemistry, wind damage, or winter damage. While the fragmentation is undesirable aesthetically and functionally, it is possible that such fragments could colonize other parts of the green roof, should they be deposited in suitable areas.

More research is necessary to determine whether this method of transplanting lichens onto green roof substrates results in viable lichen populations. Further, our transplanting method involved sourcing lichens from the wild. While these species are very common in the region, this kind of collection would clearly not be feasible on an industrial scale. Additionally, the propagation of this genus is slow. Expansion of existing colonies can occur through the fragmentation of the thallus and subsequent growth of branches that fall to the ground, vertical growth from the base, and apical growth leading to larger clumps ^19^.

Alternatives to *Cladonia* lichens providing high albedo and lack of competition with plant roots could include light-coloured gravel, stones, or woody debris, however more research is needed to determine the effect these materials would have on the green roof system. Finally, while green roofs are becoming more popular in all regions, lichens, in varying degrees, are sensitive to air pollution and many species are likely to perform poorly where air quality is low ^14^. Our study establishes that *Cladonia* lichens could provide roof cooling and water retention services in a green roof environment, but more work is necessary to explore the long-term viability of fruticose lichens in these systems.

## Figures and Tables

**Supplementary Table 1.** A soil test conducted by Nova Scotia Agriculture describing the elements present in the Soprema X 2011 substrate.

Soprema X 2011	Sample 1	Sample 2	Sample 3	Average
pH	7.7	7.7	7.7	7.7
Organic Matter (%)	5.8	6	5.9	5.9
P _2_0 _5_ (kg/ha)	669	727	739	711.7
K _2_O (kg/ha)	1606	1586	1720	1637.3
Ca (kg/ha)	4542	4860	4806	4736
Mg (kg/ha)	860	902	912	891.3
Na (kg/ha)	317	309	340	322
Sulfur (kg/ha)	463	424	552	479.7
Al (ppm)	311.85	414.78	452.07	392.9
Fe (ppm)	111	125	134	123.3
Mn (ppm)	30	34	35	33
Cu (ppm)	1.79	1.94	1.99	1.9
Zn (ppm)	6.6	6.5	6.7	6.6
B (ppm)	1.27	1.29	1.28	1.28
Nitrate - N (ppm)	41.3	33.2	44.7	39.7
% Nitrogen	0.39	0.41	0.35	0.4
Salt (mhos × 10)				
CEC (meq/100gm)	17.4	18.3	18.5	18.1
Base Sat. K (%)	9.8	9.2	9.9	9.6
Base Sat. Ca (%)	65.2	66.2	65.1	65.5
Base Sat. Mg (%)	20.6	20.5	20.6	20.6
Base Sat. Na (%)	4	3.7	4	3.9
Base Sat. H (%)	0.5	0.4	0.4	0.4
Lime Required (t/ha)	6	6	6	6

**Supplementary Figure 1.**
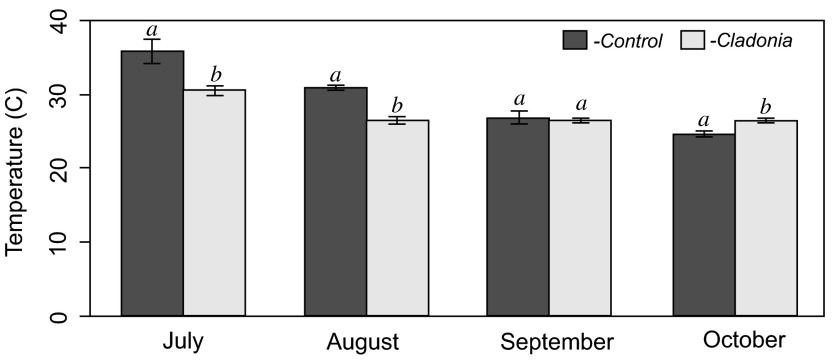
Substrate temperature for the two control modules and eight *Cladonia* modules during the 2012 growing season.

**Supplementary Figure 2.**
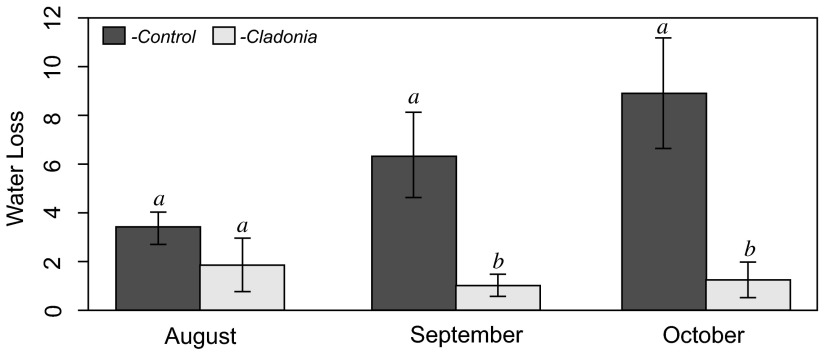
Substrate water loss for the two control modules and eight *Cladonia* modules during the 2012 growing season

**Supplementary Figure 3.**
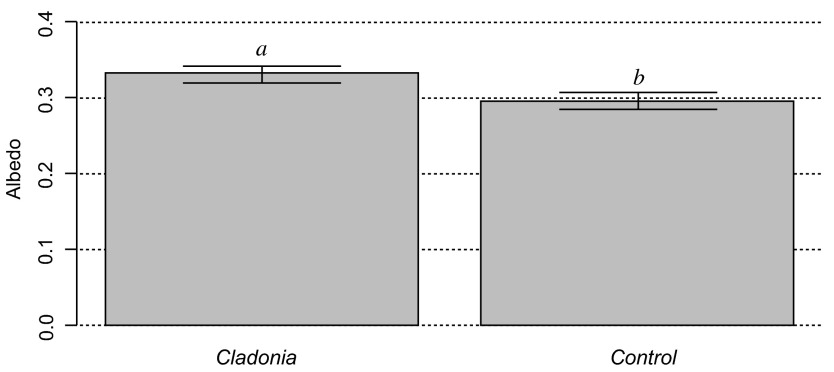
Albedo of the five control modules and five *Cladonia* modules recorded on September 28, 2013
