# Supplementary material for: Cladonia lichens on extensive green roofs: evapotranspiration, substrate temperature, and albedo
Source: F1000Res. 2014 Jan 23;2:274. Originally published 2013 Dec 16. [Version 2] doi: 10.12688/f1000research.2-274.v2 (PMC3886785; doi:10.12688/f1000research.2-274.v2)
Supplement: Peer review history

# Peer review: Referee response for version 2

**DOI:** 10.5256/f1000research.3712.r3615

**Published:** 2014-02-12

**Authors:** Richard Sutton

**Affiliations:** 1 Department of Agronomy & Horticulture, University of Nebraska-Lincoln, Lincoln, NE, USA

This revision appears acceptable to me with one minor grammatical change. The fourth sentence of the second paragraph in the Discussion section should read: " *Vascular plant species growing out of these lichen mats* ***are*** *a natural. .* . "  To get agreement between plural subject (species) and verb (is) are.

I have read this submission. I believe that I have an appropriate level of expertise to confirm that it is of an acceptable scientific standard.

# Peer review: Referee response for version 1

**DOI:** 10.5256/f1000research.3214.r2790

**Published:** 2013-12-23

**Authors:** Martin Hermy

**Affiliations:** 1 Division of Forest, Nature and Landscape, KU Leuven, Flanders, Belgium

The title and abstract cover the contents of this original work well. The study design, methods and analysis seem appropriate. Overall I do agree with the conclusions. However, one crucial element should be mentioned (which is recognized by the authors).

The fact that it is not sure that the lichens still were alive after the experiment puts considerable constraints about the applicability of these organisms in extensive green roof systems. My experience with lichens is that they normally grow on nutrient poor, dry, acidophilous soils. I've seen Cladonia's enter extensive green roof systems after a number of years (when substrate pH dropped). Green roof substrate used here (which is the standard) is indeed nutrient poor, but definitely not acid (see info). That makes the point whether these lichens are still alive or not even more important. One could eventually use dead lichen material and or other dead materials with similar properties (e.g. albedo), e.g. white pebbles.

I have read this submission. I believe that I have an appropriate level of expertise to confirm that it is of an acceptable scientific standard.

# Peer review: Referee response for version 1

**DOI:** 10.5256/f1000research.3214.r2795

**Published:** 2013-12-17

**Authors:** Richard Sutton

**Affiliations:** 1 Department of Agronomy & Horticulture, University of Nebraska-Lincoln, Lincoln, NE, USA

The Title is appropriate, the Abstract is useful and the article clearly constructed. The experimental design also appears appropriate.

I am pleased to see complete data collection information, enabling the experiment to be duplicated, however, although it is required as a part of the F1000Research format, the raw data could be more easily understood if graphed.

I have the following comments about the Conclusion/Discussion section:

 

1. The last part of last sentence in the Methods section should go into the Discussion and lead on to a more detailed discussion of the lichen’s growth (or death).
2. This following sentence is unclear. It implies that the non-planted controls had seedling growth. How could this be possible if it was simply straight substrate? *“[D]uring the trial, seedlings of trees and grasses were observed growing out of these modules, although control modules also had such seedling growth*.” How would the Sopraflora-X get seeds?
3. While beyond the scope of the study, could a line be added about transplanting/regrowth-rate/propagation? Those features have an important impact on the future use of lichens. In my experience, Lichen growth under “normal” conditions is very slow.
4. The authors should talk more about the wind damage which might be an indication that the lichens have not attached themselves. This is important because most green roofs are very windy places.
5. It is also worth noting that displaced lichen cover would function less effectively.

I have read this submission. I believe that I have an appropriate level of expertise to confirm that it is of an acceptable scientific standard.
